# Associations between brain microstructures, metabolites, and cognitive deficits during chronic HIV-1 infection of humanized mice

**DOI:** 10.1186/1750-1326-9-58

**Published:** 2014-12-18

**Authors:** Michael D Boska, Prasanta K Dash, Jaclyn Knibbe, Adrian A Epstein, Sidra P Akhter, Natasha Fields, Robin High, Edward Makarov, Stephen Bonasera, Harris A Gelbard, Larisa Y Poluektova, Howard E Gendelman, Santhi Gorantla

**Affiliations:** Department of Radiology, University of Nebraska Medical Center, Omaha, NE 68198 USA; Department of Pharmacology and Experimental Neuroscience, University of Nebraska Medical Center, Omaha, NE 68198 USA; College of Public Health, University of Nebraska Medical Center, Omaha, NE 68198 USA; Department of Internal Medicine, University of Nebraska Medical Center, Omaha, NE 68198 USA; Department of Neurology, Center for Neural Development and Disease, University of Rochester Medical Center, Rochester, NY 14642 USA

**Keywords:** ^1^H magnetic resonance spectroscopy, Behavioral and cognitive deficits, Diffusion tensor imaging, HIV-1, Humanized mice

## Abstract

**Background:**

Host-species specificity of the human immunodeficiency virus (HIV) limits pathobiologic, diagnostic and therapeutic research investigations to humans and non-human primates. The emergence of humanized mice as a model for viral infection of the nervous system has overcome such restrictions enabling research for HIV-associated end organ disease including behavioral, cognitive and neuropathologic deficits reflective of neuroAIDS. Chronic HIV-1 infection of NOD/*scid*-IL-2Rg_c_^*null*^ mice transplanted with human CD34^+^ hematopoietic stem cells (CD34-NSG) leads to persistent viremia, profound CD4^+^ T lymphocyte loss and infection of human monocyte-macrophages in the meninges and perivascular spaces. Murine cells are not infected with virus.

**Methods:**

Changes in mouse behavior were measured, starting at 8 weeks after viral infection. These were recorded coordinate with magnetic resonance spectroscopy metabolites including N-acetylaspartate (NAA), creatine and choline. Diffusion tensor magnetic resonance imaging (DTI) was recorded against multispectral immunohistochemical staining for neuronal markers that included microtubule associated protein-2 (MAP2), neurofilament (NF) and synaptophysin (SYN); for astrocyte glial fibrillary acidic protein (GFAP); and for microglial ionized calcium binding adaptor molecule 1 (Iba-1). Oligodendrocyte numbers and integrity were measured for myelin associated glycoprotein (MAG) and myelin oligodendrocyte glycoprotein (MOG) antigens.

**Results:**

Behavioral abnormalities were readily observed in HIV-1 infected mice. Longitudinal open field activity tests demonstrated lack of habituation indicating potential for memory loss and persistent anxiety in HIV-1 infected mice compared to uninfected controls. End-point NAA and creatine in the cerebral cortex increased with decreased MAG. NAA and glutamate decreased with decreased SYN and MAG. Robust inflammation reflected GFAP and Iba-1 staining intensities. DTI metrics were coordinate with deregulation of NF, Iba-1, MOG and MAG levels in the whisker barrel and MAP2, NF, MAG, MOG and SYN in the corpus callosum.

**Conclusions:**

The findings are consistent with some of the clinical, biochemical and pathobiologic features of human HIV-1 nervous system infections. This model will prove useful towards investigating the mechanisms of HIV-1 induced neuropathology and in developing novel biomarkers and therapeutic strategies for disease.

**Electronic supplementary material:**

The online version of this article (doi:10.1186/1750-1326-9-58) contains supplementary material, which is available to authorized users.

## Background

Persistent HIV-1 infection commonly leads to cognitive, behavioral and motor abnormalities called HIV-associated neurocognitive disorders (HAND) [1–3]. Despite intensive research, investigations seeking virus-associated central nervous system (CNS) biomarkers and HAND therapies have failed, in measure, due to the multifactorial nature of disease and by few relevant small animal models [4–6]. The obstacles in generating a small animal model require that viral tropism, neuroimmune activation, cognitive impairments and CD4^+^ T cell losses are operative [1, 7, 8]. This has remained an unmet goal. Added to these obstacles in mirroring human disease are the concomitant use of abuse drugs, common opportunistic infections, hepatic dysfunction, nutritional deficiencies, social demographics, ongoing antiretroviral therapies, psychiatric illness, aging and altered immune responses often seen in infected humans [9]. The shift in human disease severity, from overt dementia to subtle cognitive dysfunction, affects changes disease demographics and as such has also made modeling of human disease even more complex [10, 11]. This has occurred as a consequence of the wide spread use of antiretroviral therapy (ART) [3, 12].

Despite considerable improvements in disease severity up to half of infected patients show deficits in memory and psychomotor functions. Disease can readily be seen through neuropsychological testing using common metrics of cognitive function [13–16]. Such deficits are important as they can deeply affect the quality of life and making research into ways to find better diagnostics and therapeutic interventions timely and important [17–19]. However, in order to accomplish these goals model systems of current human disease are needed. Divergent viral-induced immune and cognitive deficits seen as a consequence of viral infection and ART need be considered. Indeed, early evidence that the terminal stages of end organ HIV disease could be reflected in rodent models of neuroAIDS was realized, over the past decade, within our own laboratories [5, 20–23]. However, despite creating such models, no cross validation of the intersection between immune and behavior abnormalities were realized. If this were possible, such a model could speed the discovery of new pathways of disease towards better understanding viral pathogenesis and even allow early diagnostic studies [5, 24, 25]. To these ends, we now demonstrate, for the first time, cross species transference of immune and viral factors that lead to neurocognitive deficits, neuropathology, brain metabolite alterations, and brain subregion damage in virus-infected NOD/*scid*-IL-2Rg_c_^*null*^ humanized mice (CD34-NSG). As such the work represents a substantive step forward from current models [22]. Remarkably, the observed metabolic [proton magnetic resonance spectroscopy (^1^H MRS)], microstructural [diffusion tensor magnetic resonance imaging (DTI)], histologic and behavioral aberrations that characterize human disease are partially replicated in the mice and as such mirror components of neuroAIDS. The model nonetheless provides unique insights in the biomolecular mechanisms of how HIV-1 infection affects neural function.

## Results

### Overview

**Figure 1:**
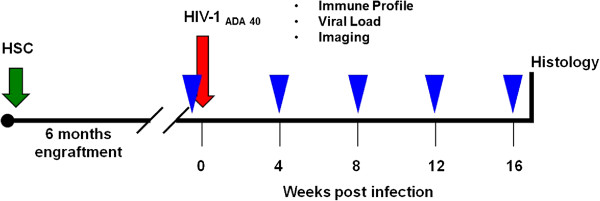
**Timings for data acquisition in combined blood, neuroimaging, and histology assessments.**

Humanized mice for studies were divided into replicate HIV-1 infected and uninfected animal groups. Imaging was performed and blood samples acquired from all animals prior to and from four weeks to 16 weeks after infection. At 16 week animals were perfused with normal saline and the brains were harvested for histopathological evaluations. The experimental scheme is outlined in Figure 1. The overall experimental design enabled cross validation of all bioimaging, viral and, immune tests with brain histopathology for each animal. In parallel experiments, mice were evaluated for behavior by open field activity (OFA). These were chosen to reflect memory and anxiety in mice. Using replicate animals for behavioral tests avoided confounding influences of the anesthesia which was required for all bioimaging tests.

### Immunologic and virologic features of HIV-1 infection in humanized mice

**Figure 2:**
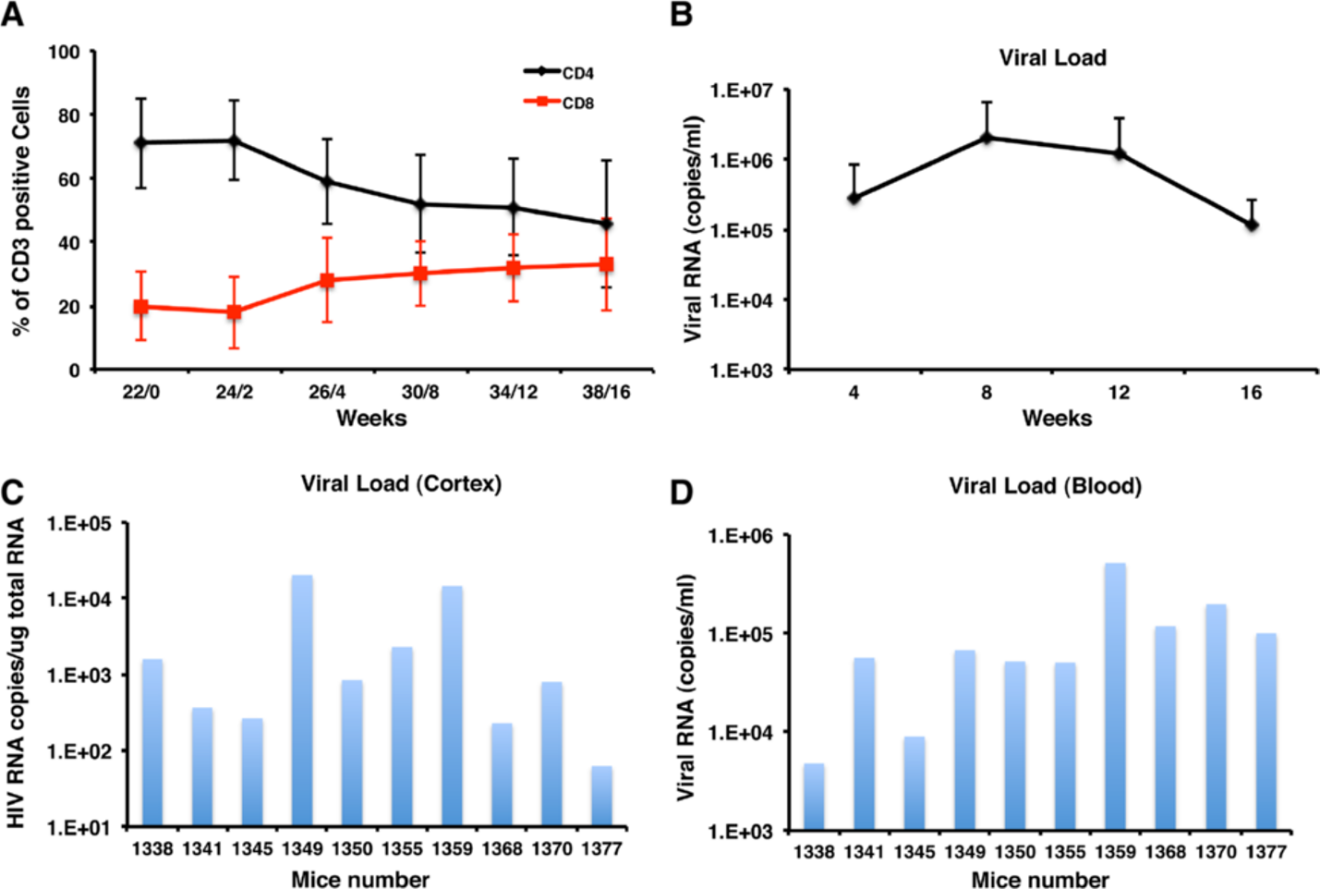
**VL and immune profiles in HIV-1 infected humanized mice. (A)** Flow cytometric analysis of CD4^+^ and CD8^+^ T cells in blood of HIV-1 infected mice (n = 10). The x-axis is the weeks following HIV-1 infection. **(B)** VL dynamics in plasma of replicate blood samples from **A**. Mice were bled once every 4 weeks starting from 2^nd^ week post-infection. Mean ± SEM are shown in both **A** and **B**. **(C)** Total HIV-1gag RNA levels in the cortex was analyzed by real time RT-PCR. Data is expressed as HIV-1 RNA copies/μg total RNA after normalizing against GAPDH (used as an internal control). **(D)** For comparison, HIV-1 viral RNA in the peripheral blood at the end point is presented as viral RNA copies/ml.

CD34-NSG mice were monitored prospectively for blood CD4^+^ and CD8^+^ T cells (Figure 2A). At 22 weeks of age, mice (n = 20) were infected with HIV-1_ADA_. VL and CD4+ and CD8+ T cells were analyzed at 4, 8, 12 and 16 weeks after infection (Figure 2B) from the 100 μl of blood collected immediately following MRS and DTI studies. Steady decreases in total CD4^+^ T cells with concomitant increases in CD8^+^ cells were observed (Figure 2A) during the course of HIV infection. Viral load (VL, copies/ml blood) peaked at eight weeks after viral infection and all infected animals had sustained VL (7.05 × 10^5^ to 1.5 × 10^7^) until 16 weeks where reductions in total human cells (as measured by CD45) mirrored decreases in VL (4.77 × 10^3^ to 5.1 × 10^6^). Replicate uninfected controls (n = 10) demonstrated stable CD45 and CD4/CD8 ratios throughout the study.

### VL and identifications of infected cells

To assess the total number of human cells and VL in the brain (copies/cc tissue), we dissected the cortex, extracted RNA, and real time RT-PCR performed for human CD45 and HIV-1gag. Comparison of VL in brains (Figure 2C) with their peripheral blood VL at 16 weeks (Figure 2D) showed that 3 animals with low peripheral VL have significant levels of HIV-1gag in brain, indicating that peripheral viral loads are not correlated to brain levels (r = 0.40, p = 0.264, Spearman correlation). The data is expressed as total HIV-1 RNA copies/μg total RNA after normalization with GAPDH. We identified the cell targets for viral infection in the periphery (spleen) and nervous system (brain). In regards to the former, HIV-1 infected CD4+ T lymphocytes and monocyte-macrophages were readily seen in both the follicular and parafollicular areas, respectively (see Additional file 1A for high power illustration). In contrast, only few numbers of infected macrophages were observed in the brain as dual labeled CD163 and HIV-1p24 cells. These were identified in the meninges and perivascular spaces, as was reported previously [5, 21] (Additional file 1B and C). This served to highlight the lack of association between viral levels and neuropathology and differences in numbers of infected cells seen in the periphery (spleen) as compared to the brain. Indeed, large numbers of infected CD4+ T lymphocytes and monocyte-macrophages were identified in the spleen.

### Behavioral deficits

**Figure 3:**
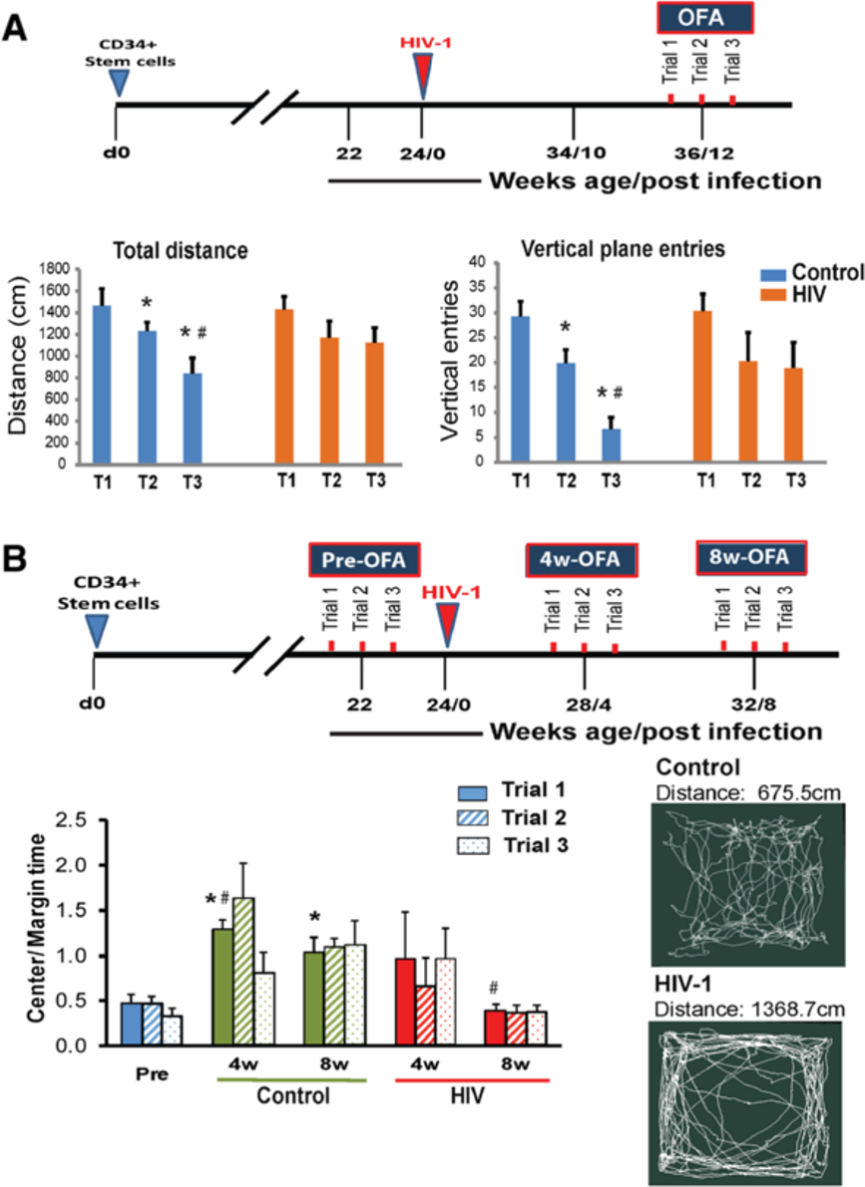
**Effect of HIV-1 infection of humanized CD34-NSG mice on cognition. (A)** Schematic diagram is illustrated showing the experimental plan used and results. Three consecutive trials of OFA testing were done at 12–13 weeks after HIV-1 infection. Replicate control mice were injected with PBS for OFA testing at the same time intervals. Mice were tested using 20 min sessions for three consecutive days (3 trials). Mice were bled from sub-mandibular vein under isoflurane inhalation anesthesia for flow cytometry and VL measurements. Total distance travelled measured in the floor plane and vertical entries were measured in the vertical plane. These reflect the exploratory and habituation behavior of the mice in a new environment. Both measurements were reduced by the 2^nd^ and 3^rd^ trials in uninfected controls reflecting habituation. HIV-1 infected animals exhibit continued anxiety. *, p < 0.05 compared to 1^st^ trial and #, p < 0.05 compared to 2^nd^ trial. Values are mean ± SEM. **(B)** OFA testing was performed longitudinally before infection and 4 and 8 weeks after HIV-1 infection (n = 8 for both control and infected animals). At each time point mice were tested for OFA using 20 min session for three consecutive days (3 trials). Time spent and distance traveled in the center compared to the periphery was automatically measured. The ratios were analyzed to assess anxiety behavior and long term memory of environment. By 8 weeks, control mice showed memory of the environment and reduced anxiety by spending more time in the central bright zone compared to HIV-1 infected mice. * = p < 0.05 compared to trial 1 of pre-infection OFA, and # = p < 0.05 compared to the corresponding trial performed at the same time from controls. Values are mean ± SEM. (**B**, bottom right) First five minute travel paths with distance travelled for representative control and HIV-1 infected mice from trial 3 of an 8 week time point are shown.

The behavioral phenotypes of the HIV-1 infected humanized CD34-NSG mice were compared to uninfected controls by OFA testing [26–28] in a replicate mice. Here, mice are placed in an enclosure that permits exploration of a new environment. Spatial distribution, horizontal and vertical movements are then measured to assess exploratory behaviors. By the third trial, reductions in total horizontal distance and vertical movements in uninfected controls reflected mouse habituation associated with loss of anxiety and memory of the environment [27, 29]. Unlike uninfected controls, HIV-1 infected animals showed unremitting activity even at the third trial demonstrating continued anxiety (Figure 3A). Implementing OFA measurements over successive trials at monthly intervals permitted investigation of the level of memory with HIV-1 infection (Figure 3B). Analysis of central zone activities, expressed as the ratio between times spent in the margin to total time (Figure 3B) indicated significant differences between animal groups at 4 and 8 weeks after repeated testing and infection. Control animals demonstrated memory of the environment that is not seen in the infected animal groups. Measures of central/total time 4 and 8 weeks demonstrated that loss of anxiety readily occurred in uninfected mice but not HIV-1 infected animals. These animals showed continued anxiety by thigmotaxis [30]. These tests best assess exploratory behavior, locomotor activity and anxiety-like behavior and were employed based on the fragile nature of the animals that would not tolerate the more rigorous Morris water maze testing.

### Multispectral fluorescence imaging of immunohistology

**Figure 4:**
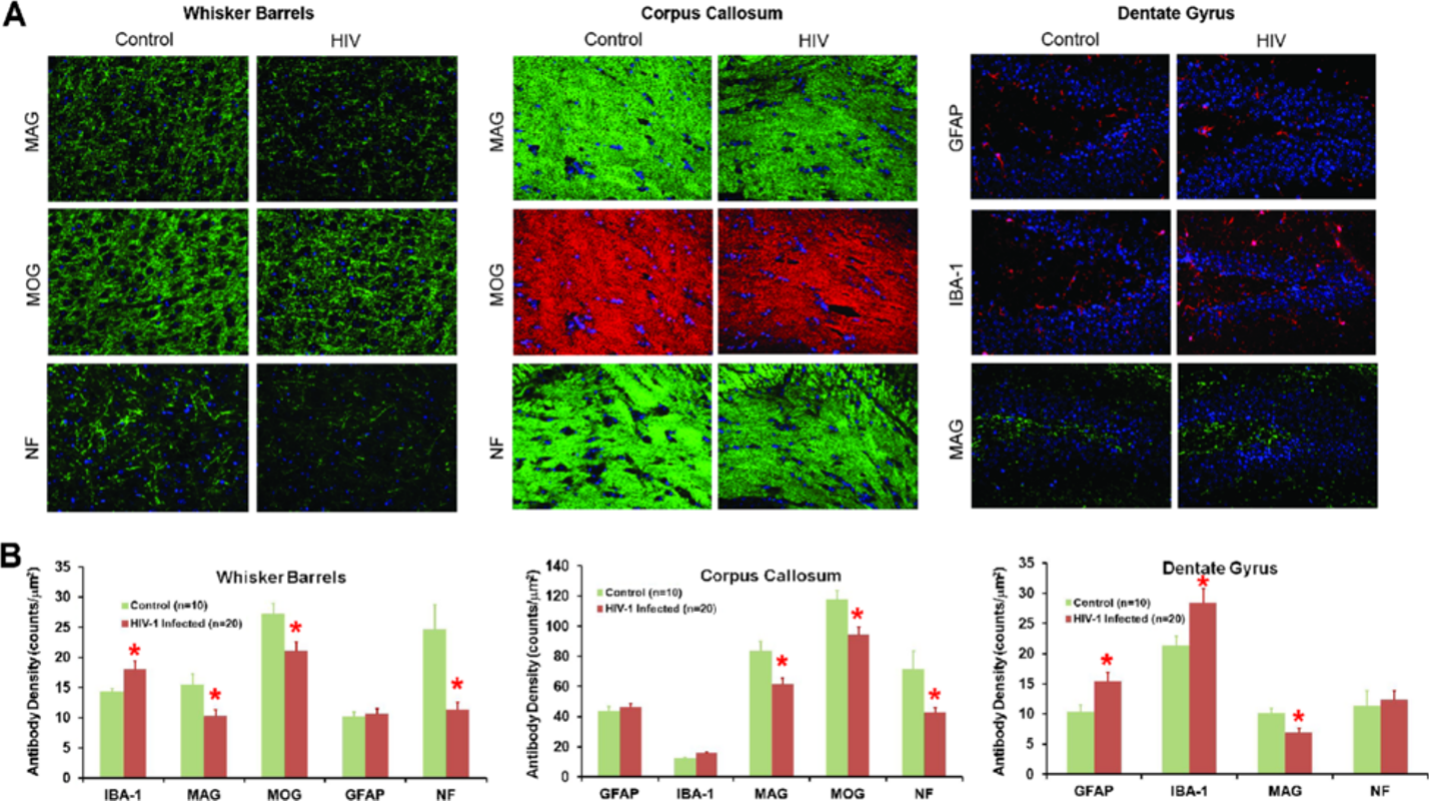
**Immunofluorescence staining of neuronal and glial antigens following HIV-1 infection.** Paraffin embedded 5 μM brain sections were immunostained with MAP2, SYN, NF, GFAP, MAG and Iba1 antibodies. **(A)** Representative images captured at 40× magnification from whisker barrel (WB), corpus collosum (CC) and Dentate gyrus, (DG) are shown for those markers significantly deregulated after HIV-1 infection. **(B)** Density of antigen expression quantified in different brain regions are shown for both HIV-infected and uninfected mice by multispectral imaging. Decreased expression of MAG and NF in WB, CC and DG regions of infected animals compared to uninfected controls. Significant increase in reactive microglia and astroglyosis (GFAP) was observed in DG. Oligodendrocyte associated myelin (MOG) was reduced both in WB and CC. Genu of corpus callosum from infected animals show decreased expression of MAG, MOG and NF in infected mice. Values are mean ± SEM and *denotes p < 0.05.

Neuronal and glial antigens were analyzed by multispectral fluorescence imaging of replicate brain regions at study termination. Images of each fluorescent spectral component were analyzed following staining. Neuronal density was assessed using neuronal microtubule associated protein-2 (MAP2) and neurofilament (NF). Synaptic density was assessed using synaptophysin (SYN). Activation of astrocytes was assessed using glial fibrillary acidic protein (GFAP). Microglial activation was measured using ionized calcium binding adaptor molecule 1 (Iba-1). Oligodendrocyte numbers and integrity were measured by myelin associated glycoprotein (MAG) and myelin oligodendrocyte glycoprotein (MOG) antigens. (Figure 4). These antigens are expressed as fluorescence intensity per μm^2^ (Figure 4B). Data from 20 infected animals were compared to 10 uninfected controls.

SYN expression showed punctate and diffuse distribution in cortical areas of control animals, but in case of HIV-1 infected animals, SYN labeled regions were irregularly shaped with significantly decreased expression and fluorescence intensity in the M2 region of the cortex (Additional file 2) with a trend towards decreased SYN in the whisker barrels (Figure 4B). No significant altered expressions of SYN were seen in the 4 subregions of hippocampus. GFAP expression was increased in 3 regions of hippocampus (CA1, CA3 (Additional file 2) and DG (Figure 4)). This reflects astrocyte activation responses that commonly follow HIV-1 infection. Microglial expression, measured by Iba-1 staining, increased in the M2 region of the cortex (Additional file 2), the whisker barrels, and the dentate gyrus (Figure 4). In contrast, significant reductions in NF-positive fibers were observed in the whisker barrels and corpus callosum of HIV-1 infected mice (Figure 4) while changes in NF expression between infected and uninfected brains were not seen in the hippocampus (Additional file 2). These results, taken together, demonstrate that HIV-1 infected humanized mice are distinct from previous injection models of HIV-1 encephalitis where glial inflammatory responses drive more significant neurodegeneration [5, 23]. Nonetheless, the reduced presynaptic and neurofilament expression seen in the cortical and white matter regions of the humanized virus-infected mice indicate ongoing neurodegeneration potentially compatible with an orthograde axonopathy.

We next analyzed MOG and MAG in the white and grey matter regions to assess oligodendrocyte and myelin density. MOG, is a myelin component of the CNS and responsible for maintenance of the myelin sheath, cell adhesion and oligodendrocyte microtubule stability. MOG is localized on the oligodendrocyte cell surface and on the outermost lamellae of mature myelin. MAG is a nervous system cell surface adhesion protein that is involved in linking myelinated cells to neuronal axons. Like myelin, MAG inhibits axonal outgrowth and contributes to the inhibitory properties of myelin. We observed significantly decreased MAG expression in the whisker barrels, corpus callosum, and hippocampus regions DG (Figure 4), CA1, and CA3 (Additional file 2) in HIV infected animals. Decreased MOG expression was observed in the whisker barrels and corpus callosum of infected animals (Figure 4) suggesting loss of axonal fiber elements responsible for HIV-1 associated grey and white matter damage.

### Correlations between antigens and viral load (VL)

Mean results provide a picture of damage focused in the cortex and white matter tracts. Hippocampus is affected primarily with inflammation. The degree of damage is associated, in measure, with the mean virus concentration in the blood over the course of infection. We hypothesized that variability between individual mice infection level (VL range 5 × 10^4^ to 6 × 10^6^ copies/ml blood) would lead to differing severities of neuronal disease as reflected by quantitative histological measures. Surprisingly, there were no significant correlations between the virus levels and the quantitative immunohistology. Only trends between MAG reductions in the corpus callosum (r = -0.386, p = 0.1) and GFAP in the dentate gyrus (r = -0.399, p = 0.098) were found.

### Neuroimaging

Within a subset of the animals studied we acquired ^1^H MRS and DTI at four week intervals from preinfection, to 16 weeks post infection in both infected animals (n = 8) and uninfected humanized NSG mice as controls (n = 7). In addition to comparing the groups over time to determine the kinetics of brain pathology in this model, results for the individual infected animals were tested for correlations to virus levels and histopathology to aid in the interpretation of changes observed.

### ^1^H MRS

**Figure 5:**
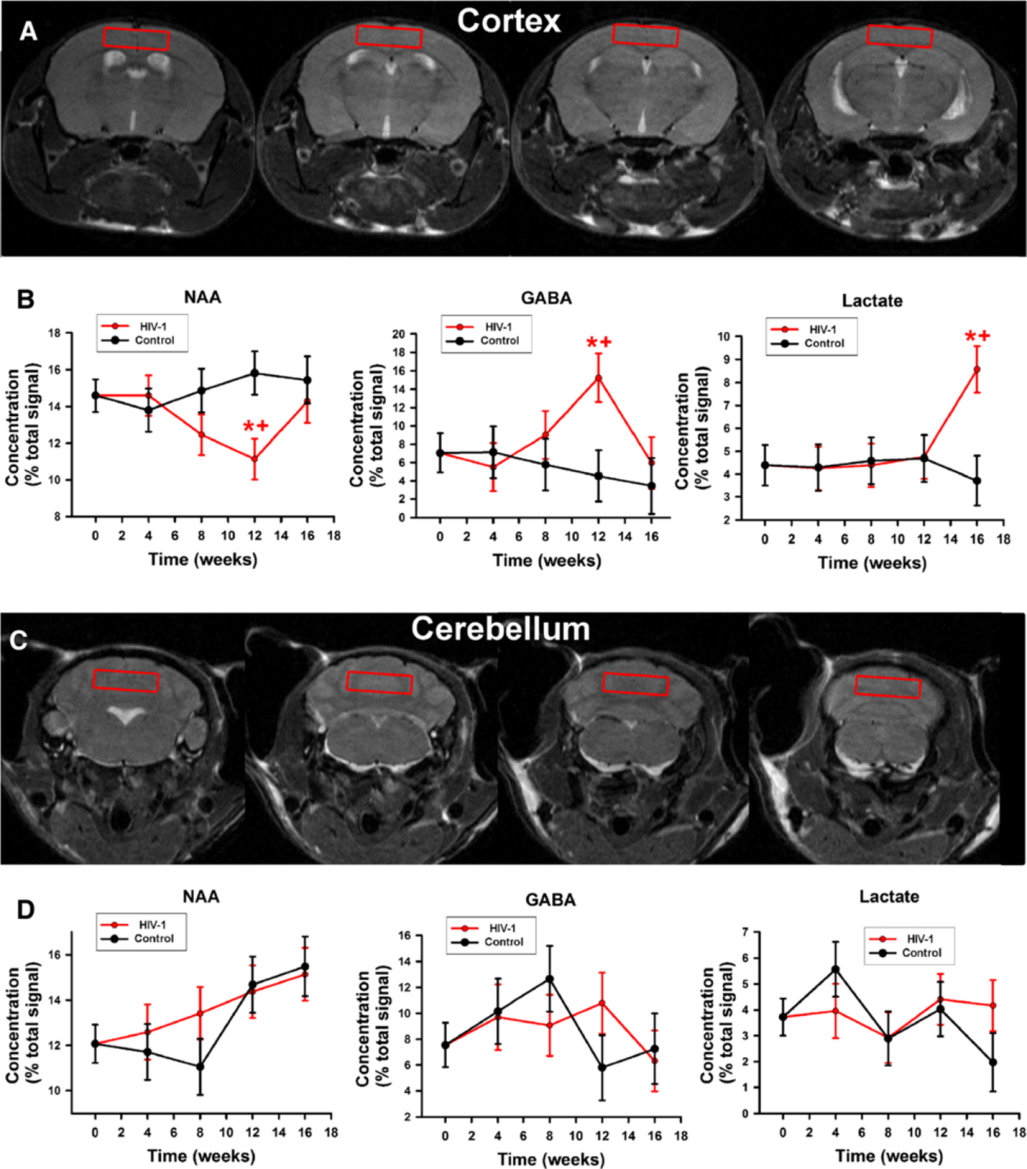
**Metabolite levels (Means ± SEM) expressed as a percentage of total signal acquired over time from** ^**1**^ **H MRS scans of (red) HIV-1 infected (n = 8) and (black) uninfected humanized mouse controls (n = 7). (A)** Region selected for spectral acquisition from the cerebral cortex. **(B)** Selected metabolite levels are measured from the cerebral cortex. Time zero, in infected mice is before infection with subsequent spectra acquired every four weeks to 16 weeks in both infected and control mice. **(C)** Region selected for acquisition of spectrum from cerebellum. **(D)** The same metabolites as shown in **(B)**. Time courses of all metabolites are shown in Additional files 3 and 4. *p < 0.05 control versus HIV-1 infected mice, +p < 0.05 vs time zero in control mice, (red “+” symbol) p < 0.05 versus preinfection in infected mice.

Mean metabolic consequences of HIV-1 infection as compared to uninfected animals can be seen in the cerebral cortex (Figure 5A, B). Reduced NAA and increased GABA were seen at 12 weeks and reversed at 16 weeks in the cerebral cortex but not in the cerebellum (Figure 5C, D). Notably, a large increase in lactate was observed in the cerebral cortex at 16 weeks after infection (Figure 5A, B). Other metabolic effects of infection include reductions of creatine and choline in the infected cerebral cortex over time (Additional file 3) which is not seen in the cerebellum (Additional file 4). These changes are subtle and not significantly different from what is generally observed in uninfected animals. Indeed, in the cerebral cortex of infected animals the reductions of choline and creatine were significantly different only from the preinfection levels of the same animals.

### Correlations between cortical metabolites and VL

To further examine the relationship between blood virus levels and brain pathology, we investigated the correlations between end-stage (16 week post infection) metabolite concentrations and the mean blood virus level over the course of infection. Only one of the metabolites had a significant correlation with mean virus concentration, lactate (r = 0.857, p = 0.024) with a trend towards correlation with myoinostitol (r = -0.714, p = 0.088). While increasing lactate would be expected with higher viral loads, decreasing myoinostitol with higher viral loads is unexpected, as increased neuroinflammation is presumed to be associated with increased myoinostitol. In order to further explore these effects, we investigated the correlations between 16 week post infection metabolite concentrations and quantitative immunohistology in the cerebral cortex.

### ^1^H MRS cortical metabolites and neuronal, oligodendrocyte and glial markers

**Figure 6:**
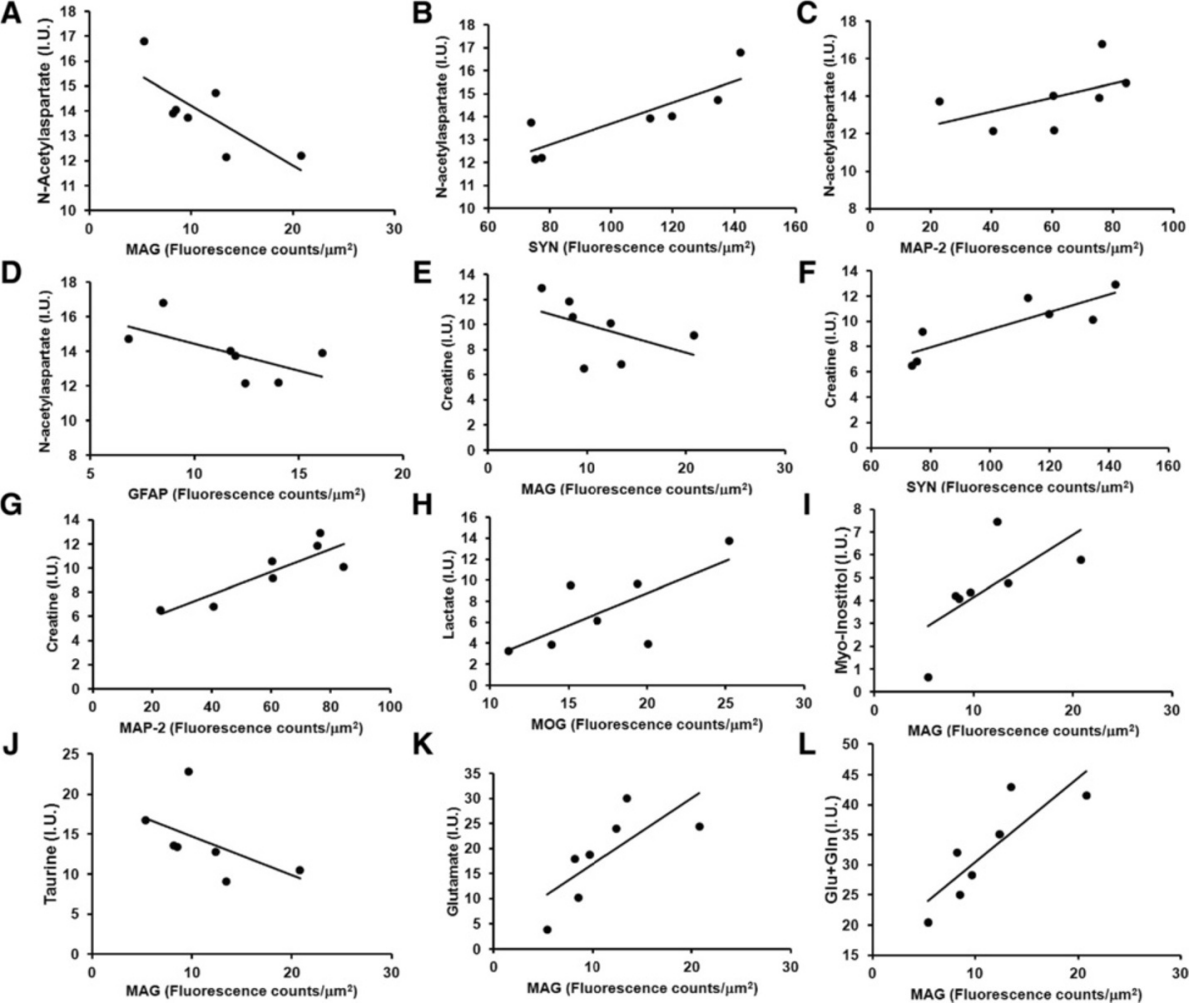
**Correlations between cortical metabolites at 16 weeks after infection and quantitative multispectral histology.** The latter was measured in the M2 region of the cerebral cortex. **(A-D)** NAA versus MAG, SYN, MAP2 and GFAP respectively are shown. **(E-G)** Creatine versus MAG, SYN, and MAP2 are illustrated. **(H)** Lactate versus MOG. **(I-L)** MAG versus myoinostitol, taurine, glutamate, and glutamate and glutamine, respectively were analyzed in these data sets.

The correlations between metabolite concentrations at the end-point of the study with cortical markers for synaptic density (SYN), glial activation (Iba-1, GFAP), and oligodendrocyte-associated proteins density (MAG, MOG) were measured between the cortex spectrum and the quantitative histology from the M2 region of the cortex. Significant correlations were found between reduced MAG in the M2 region of the cerebral cortex (M2c) and reduced 16 week concentrations of the metabolites glutamate (r = 0.929, p = 0.024), myoinostitol (r = 0.857, p = 0.024) as well as a trend for increased taurine (r = -0.750, p = 0.066) (Figure 6I-K). In addition, there was a trend for increased NAA (r = -0.714, p = 0.088) and creatine (r = -0.750, p = 0.066) to correlate with reduced M2c MAG (Figure 6A, E, I-L). Similar, but opposite relationships existed between SYN and NAA (r = 0.893, p = 0.012), and creatine (r = 0.857, p = 0.024) (Figure 6B, F). In addition, there was a tendency for increased GFAP levels to correlate with reduced NAA (r = -0.714, p = 0.088) (Figure 6D). Finally, it was also found that levels of lactic acid showed a trend towards correlating with MOG (r = 0.750, p = 0.066) (Figure 6H). This provides an overall picture of cortical metabolism changes from HIV-1 infection showing that processes leading to synaptic loss are associated with loss of NAA and creatine, neuroinflammation being associated with NAA loss, while reduced MAG are associated with increases in NAA, creatine, taurine, and reductions in glutamate and myoinostitol. Certainly, these associations cannot be over interpreted, but are generally in agreement with expected metabolic effects of inflammation, synaptic loss, and loss of oligodendrocytes-associated proteins in the cortex of these infected animals.

### DTI

**Figure 7:**
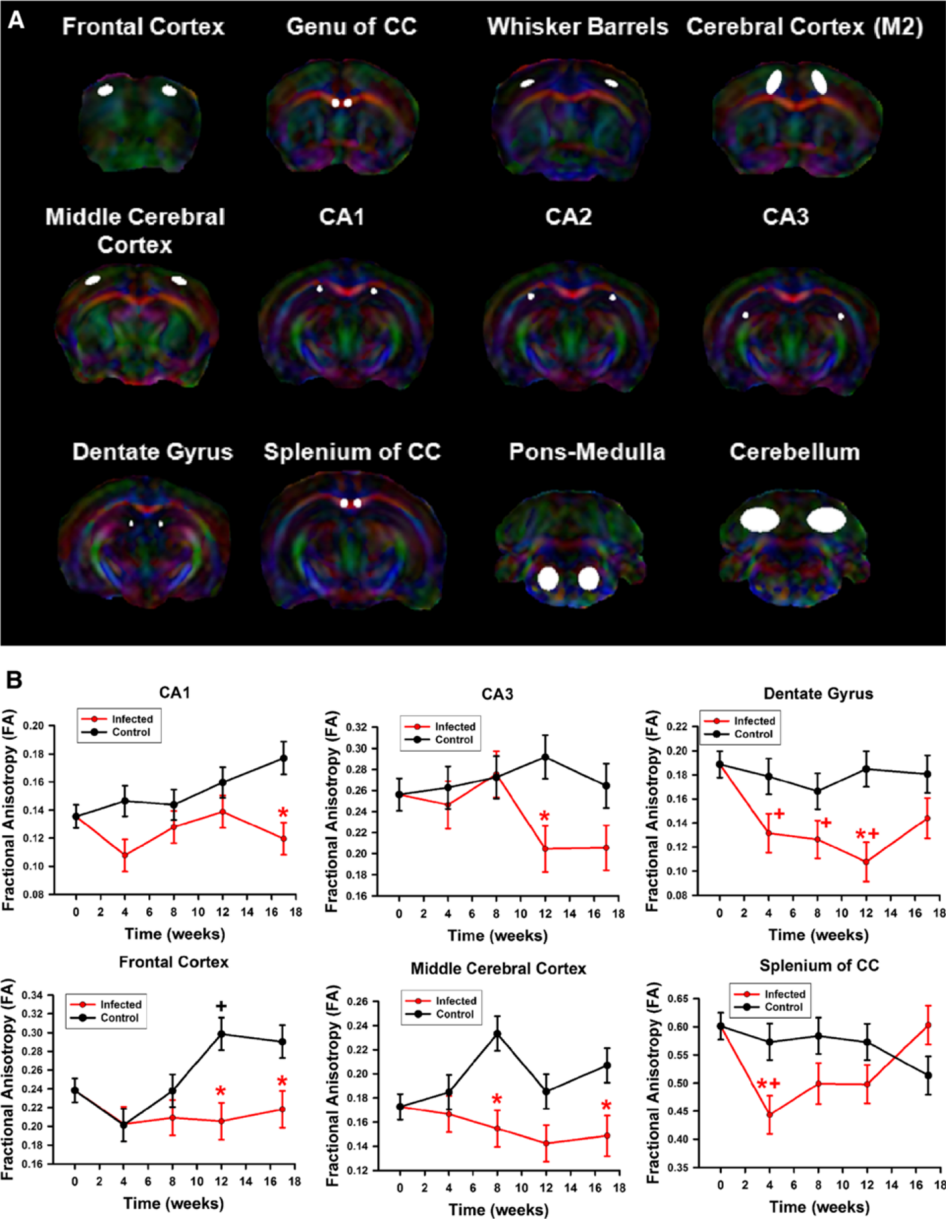
**DTI metrics. (A)** Brain regions analyzed for DTI metrics. **(B)** Fractional anisotropy (mean ± SEM) in (top) CA1, CA3, and dentate gyrus (from left to right) and (bottom) frontal cortex, middle cerebral cortex, and splenium of the corpus callosum (from left to right) as shown in **(A)**. (red “*” symbol) p < 0.05 control (n = 7) vs infected (n = 8) mice, ^+^p < 0.05 versus time zero in control mice, (red “+” symbol) p < 0.05 versus pre-infection in HIV-1 infected animals.

DTI metrics investigated include FA, D_av_, longitudinal diffusivity (λ_l_) and transverse diffusivity (λ_t_) (see Materials and Methods) in brain regions. These were measured in the hippocampus [CA1, CA2, CA3 and dentate gyrus (DG)], in cortex (whisker barrels, middle cerebral cortex, M2 cortical region, frontal cortex), and white matter regions (splenium and genu of the corpus callosum) (Figure 7A). Metrics of uninfected control and HIV-1 infected animals were compared over time. Results of individual mice and brain regions were evaluated independently with correction for multiple comparisons. The most consistent and notable changes were seen as reduced FA in hippocampus and cortical regions relative to the uninfected controls (Figure 7B). Many of these differences are accompanied by increase in FA in the uninfected humanized mice that did not manifest in the infected mice. It is also notable that the cause(s) of such a reduction in FA in some regions are reversible, including the dentate gyrus and splenium of the corpus callosum, possibly indicating transient inflammatory processes occurring in these regions during the course of infection. Other notable alterations included increased diffusivity in white matter (Additional files 5, 6 and 7).

### DTI and VL

**Figure 8:**
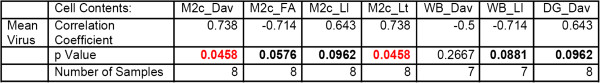
**Correlations between cortical DTI metrics and mean plasma VL 16 weeks after HIV-1 infection.**

End-point values of DTI metrics were tested for correlation with mean viral loads in the individual infected mice (Figure 8). Correlations with virus level were weak and found only in cortical regions (M2 region of the cortex and whisker barrels) in multiple DTI measures, but only a trend of a single correspondence in DG and no other regions. This suggests that the cortical regions are the primary site of neuronal damage due to multifactorial HIV-1 induced injuries. Exactly which factors are primary causes of this damage will be a matter for further exploration. Nevertheless, further details regarding specific histological abnormalities and DTI metrics were then explored in the cortical regions, hippocampus, and corpus callosum.

### DTI and neuronal, oligodendrocyte and glial markers

**Figure 9:**
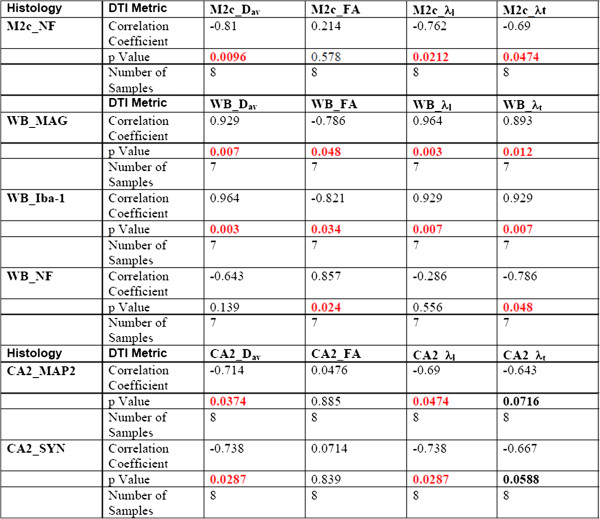
**Correlations between DTI metrics and histological quantifications in cortex and corpus callosum.**

End-point values of DTI metrics were correlated with quantitative histopathology in the individual infected mice (Figure 9). Correlations were considered between the DTI metrics within spatial subregions used for histological analysis which included cortical regions (M2 region of the cortex (M2c), whisker barrel regions (WB)), hippocampus regions (CA1, CA2, CA3 and the dentate gyrus (DG)), genu of the corpus callosum, cerebellum, and pons-medulla (brainstem) as delineated in Figure 7. The most significant correlations were found in the cortical regions, similar to virus level. It was observed that in the whisker barrel region increasing mean diffusivity and decreasing FA correlate with increasing MAG and with increasing Iba-1. In addition, FA reduced with NF loss. The CA2 region of the hippocampus shows correlation of increased diffusivity, especially the longitudinal diffusivity with decreasing SYN with a similar trend with MAP2. No significant correlations were found in the corpus callosum, brainstem, or the cerebellum.

## Discussion

There are expected similarities and noted differences between the neurological consequences of HIV-1 infection in humans and in this mouse model. As for the former, chronic infection in the mice leads to viral invasion of the central nervous system and ingress of monocyte-derived macrophages first into the meninges then the perivascular spaces. Behavioral abnormalities follow but more subtle in mice. They are coincident with CD4+ T cell loss and high peripheral viral loads. Notably, mouse infection leads to micro- and astro- gliosis and neuronal loss that, like in humans, are region and animal specific. Indeed, levels of infection and histopathological findings in brain vary. Such variability rests in the extent of inflammation and neuronal damage. We have not yet observed a true multinucleated giant cell encephalitis in mice as is often present in humans and likely due to that primary microglia and astrocytes are rodent-in-origin and as such cannot be infected with the virus. Nonetheless, the finding in these rodents underlies the central tenet of human disease in that few infected cells in the brain can give rise to more wide spread alterations in neural function underpinning the role of a virus-induced metabolic encephalopthy. Importantly, it is the engagement of the innate and adaptive immune system triggered by the virus that are not only operative in the infected reconstituted mice but the best reflection of human disease [5]. While this model replicates many of the features of human disease, limitations exist. These include the need for handling the animals in a P3 facility, the extensive work required for humanization, and the sterilizing equipment and procedures necessary. In addition, as is known the virus infects only human cells. Thus the feature of infected microglia and astrocytes within the brain parenchyma are not replicated. Only peripheral monocytes and macrophages and CD4+ T lymphocytes migrating across the blood-brain barrier are infected and that infection is limited to the meninges and perivascular spaces [5, 21]. The relatively few infected cells are a likely source for paracrine inflammatory responses that affect more wide spread neural injuries. This serves, in part, to explain the disconnection between absolute virus levels and neuropathology. Additional advantages of this humanized mouse model over more widely used transgenic mice that express viral proteins or injection of HIV-1 infected cells into brain areas affected by disease is that the current model can accurately reflect ongoing viral infections rather that specific virotoxins or acutely generated cellular inflammatory factors [5, 31]. The model is thus a significant step forward in studies of HIV-1 neuropathogenesis as relevant neuroAIDS models were previously only available in nonhuman primates [32, 33]. These were costly and due to differences in viral dynamics did not always mimic natural progression of CNS dysfunction. Thus, we developed this model as one possible solution. The new model is based on the fact that immunodeficient mice transplanted with human hematopoietic CD34^+^ stem cells (HSC) (hu-NSG) at birth enable long-term engraftment of a functional human immune system and support chronic HIV-1 infection [21, 22, 34]. Whether this could lead to CNS consequences of persistent infections and the types of behavioral, histologic and imaging abnormalities common in infected humans was not known. Our findings resolve such an unknown, in part, by demonstrating that systemic HIV-1 infection leads to CNS damage and providing opportunities to detail brain metabolic dysfunctions [35]. Interestingly, relationships between levels of viral replication and virus-associated pathology did not uniformly occur. As ART is not always able to penetrate the blood-brain barrier permitting reservoirs for viral mutation; HIV infection of glia (astrocytes, microglia and blood borne macrophages) persists in humans [36–38].

We had originally thought that viral load would be the major determinant of neuronal dysfunction in the CD34-NSG mice. This proved wrong. It is the lack of correlation between virus levels and neurodegeneraation that demonstrates that, as in humans, the mechanisms of neuronal damage are complex and not simply a matter of absolute viral load. While a clear hypothesis of what does cause neuronal damage is not possible using the present results, we find that the time course of chronic peripheral infection in the mice from the neuroimaging results indicates initial inflammation, followed by neurodegeneration and subsequent oligodendrocyte degeneration. Factors influencing this progression likely include the pathobiologic details of infected immune cells and the effects of cytokine and chemokine release on neuronal function. The ability to explore these factors will be the subject of future studies and likely will result in the ability to identify specific mechanisms involved in HIV-1 neurocognitive dysfunction.

Developing a database of co-registered histology, DTI, and metabolic changes along with clinical indications including CD4/CD8 and virus levels, and in the future, protein, chemokine, cytokine and metabolomics, will aid in our abilities to develop non-invasive monitors of disease status and understand the mechanisms of brain reaction to peripheral infection even with relatively few infected cells in brain.

Histological analyses demonstrated links between peripheral viral infection and cortical neuroinflammation, synaptic and neuronal loss, and damaged oligodendrocytes. This was seen especially in the whisker barrels. Not surprisingly, NAA was reduced during chronic infection with neuronal damage and neuroinflammation. Also, increased NAA correlates with oligodendrocytes dysfunction at the endpoint of the 16 week time course of infection. Oligodendrocytes break down NAA to acetate and aspartate via the aspartoacylase enzyme. In a recent report oligodendrocytes have been shown to play an important role along with neurons and astrocytes to form a tri-cellular compartment [39]. Glial and neuronal cells communicate by interchange of NAA, with neuronal NAA formation in synaptosomal mitochndria [40], oligodendrocytes breakdown of NAA to provide acetyl groups for myelin synthesis [41], and release aspartate and acetate to be taken up by neurons for resynthesis of NAA. The time course of NAA in infected animals suggests that inflammation and neuronal damage start early in infection, with oligodendrocyte dysfunction and loss occurring later in the time course of infection in this animal model. It was also found that creatine shows a similar correlation as neuronal damage correlates with decreased creatine and oligodendrocytes dysfunction correlates with a creatine increase. Dysfunction of oligodendrocytes have been seen in human HIV-1 infection as global reduction in myelination, reflected by reduced MRI magnetization transfer [42]. Creatine increase has been shown in the white matter during acute stages of accelerated simian immunodeficiency virus infection [43] and similarly increased in the frontal white matter of antiretroviral naïve HIV+ humans while decreasing in deep gray matter (basal ganglia) in relation to dementia severity [44]. Creatine reduction has been shown in some neurodegenerative disorders using either internal water signal or total metabolite concentrations as a normalization factor. Creatine reduction has been demonstrated in mild cognitive impairment [45] and in the prefrontal cortex of adults with autism spectrum disorders [46].

Correlations between DTI and histology provide clues to viral neuropathogenic mechanisms. Increased diffusivity and decreased fractional anisotropy were associated with both neuroinflammation and neuronal damage. Neuronal damage is mostly associated with increased transverse diffusivity, as opposed to gliosis which correlated with mean diffusivity. Conversely, loss of oligodendrocyte function is associated with decreased diffusivity and increased fractional anisotropy. These findings are clearly linked to MAG distribution and staining intensities in the infected animals. MAG may serve as a receptor for neuronal ligand(s) that modulate glial responses. The increase in activated microglia in the somatosensory cortex and white matter may be responsible for the NF and presynaptic reductions seen mediated through neurotoxic factors.

The gold standard in the diagnosis of HAND is neuropsychological abnormalities [47, 48]. We demonstrated impaired ability to recognize and habituate to a new environment following HIV-1 infection. Cognitive deficits were also seen in mice injected intracranially with HIV-1 infected human macrophages and transgenic animals expressing HIV-1 proteins [49–54]. These abnormalities were more significant than what was seen in the current model likely due to the concentration of virotoxins and the resultant high degree of inflammation [49, 55]. NF loss in cortical regions and increase in astrocyte activation in hippocampus as well as altered metabolites in these regions may also contribute to the behavioral abnormalities and memory loss. Moreover, the behavioral changes observed in our mice (anxiety and memory loss) parallel some of the dysfunctions reported by the National Institute on Mental Health cohort followed by the CNS HIV Anti-Retroviral Therapy Effects Research (CHARTER) group. These studies used standardized neuropsychological tests. ART-associated improvements in verbal fluency, information processing speeds, and motor domain function were noted [56]. Interestingly, patients continued to show mild deficits in learning and memory and executive functions despite ART [3, 57]. Whether changes in the cortical impairment link to specific pathobiological outcomes remain of considerable interest [3]. Changes in synaptic plasticity by neuroinflammation resulted in subtle memory losses with habituation and demonstration of anxiety with HIV-1 infection attributing to decreased hippocampal integrity [58]. Interestingly, reduction in creatine levels following creatine kinase knock out led to reduced hippocampal fiber formation and deficit in open field habituation [59]. Evidence of such behavioral abnormalities in this mouse model of chronic infection strengthens its relevance in mirroring some aspects of the human disease.

Persistent systemic viral replication affects memory and lead to anxiety, neuroinflammation and defects in neural metabolism [60–64]. The notable features of HIV neuropathogenesis include infection of the nervous system by invading leukocytes that traverse the blood-brain-barrier with blood borne perivascular macrophages and microglia infection. Glial activation, white matter pallor, neuronal injury and subsequent neuronal death occur through exposure to viral proteins and cellular neurotoxic factors [65]. These are also seen in HIV infected immune deficient mice [23]. Neuronal injuries may be sped by active viral replication in peripheral lymphoid tissues [66].

## Conclusions

In conclusion, we provide evidence that this murine model of viral infection produces neurocognitive dysfunction as a consequence of systemic HIV-1 infection. This model will be useful for investigating mechanisms of HIV-1 neuropathogenesis. The combination of behavioral tests, advanced neuroimaging, and histology are a powerful combination that is best used to detail some of the neuropathological processes caused by HIV-1 infection.

## Methods

### HIV infection of humanized mice

NOD/*scid*-IL-2Rg_c_^*null*^ (NSG) mice obtained from Jackson Laboratories established breeding colonies (stock number 005557) and housed under pathogen-free conditions; done in approval and in accordance with ethical guidelines for care of laboratory animals as set forth by National Institute of Health and the University of Nebraska Medical Center (UNMC IACUC 06-071-02FC). Human CD34+ hematopoietic stem cells (HSC) were obtained from fetal liver (University of Washington, Laboratory of Developmental Biology, supported by NIH award 5R24HD000836) by magnetic bead selection (Miltenyi Biotech Inc., Auburn, CA), and NSG mice were humanized as described previously [20]. Human immune system engraftment was determined by flow cytometry in rodent peripheral blood using antibodies to human pan-CD45, CD3, CD4, CD8, CD14 and CD19 in a six-color combination (BD Pharmingen, San Diego, CA). The percentages of CD4^+^ and CD8^+^ T-lymphocytes were obtained from the gate set on human CD3^+^ cells. Humanized CD34-NSG mice were infected intraperitoneally with HIV-1_ADA_, a CCR5 utilizing virus at a dosage of 10^4^ tissue culture infective dose 50 (TCID_50_)/mouse at 22 weeks of age. The levels of viral replication were monitored and analyzed from plasma at defined time points by an automated COBAS Amplicor System V1.5 (Roche Molecular Diagnostics) and expressed as copies of viral RNA per ml sample (Viral load, VL) as previously described [22]. HIV-1_ADA_ used for infection was propagated in monocyte-derived macrophages [67].

### Real-time RT PCR

Total RNA from cortex was extracted using TRIzol (Invitrogen, Carlsbad, CA) method. Recovered RNAs were reverse transcribed to cDNA with random hexamers (Invitrogen). Real-time quantitative PCR was performed using an ABI PRISM 7000 sequence detector (Applied Biosystems, Foster City, CA). Human CD45, and mouse GAPDH expressions were analyzed using TaqMan gene expression assays. For HIV-1 gag the primers and probe used were: forward, 5′-ACATCAAGCAGCCATGCAAAT-3′; and reverse, 5′-ATCTGGCCTGGTGCAATAGG-3′ and probe (FAM), 5′-CATCAATGAGGAAGCT GCAGAATGGGATAGA-3′ (TAMRA). Viral copies were calculated from the standard curve using DNA from the 8E5 cell line (NIH AIDS reference reagent program).

### Behavioral testing

Behavioral testing was performed in a dedicated series of animals that were not subjected to monthly neuroimaging evaluations. Humanized mice were subjected to an open field activity (OFA) testing after HIV-1 infection. Control mice (n = 9) were left uninfected but tested at the same age as infected mice (n = 7). OFA testing was conducted using a TruScan automated photo beam-tracking arena (Coulbourn Instruments, Lehigh Valley, PA). The arena is equipped with two photo beams, the first one measures the beam breaks in x and y axes closer to the floor and the second measures movements in z-plane. The number of horizontal and vertical beam breaks assessed horizontal and vertical activity, respectively. Animals were acclimated to the experimental room for at least 1 hour prior to testing. Mice were individually placed in the center of a 25.4 × 25.4 cm square arena and left to freely explore for 20 minutes. Parameters were automatically monitored and measured by TruScan 2.02 software, which included total distance travelled (ambulatory), velocity, ambulatory time, rearing and vertical movements. OFA testing was performed over three consecutive days in the same arena to measure habituation to the new environment. All trials were conducted at the same time of the day (from 13:00 to 18:00 hours). The arena was cleaned before testing each animal using 70% ethanol to remove any olfactory cues of human or mouse origin. For longitudinal OFA using a new set of mice, testing was done at pre-infection and 4 and 8 weeks post infection. At each time point, trials were performed on three consecutive days. The arena was divided into darker marginal zone, which is a 2.5-beam margin of the walls and brighter central zone. Time spent and distance travelled in the center compared to the periphery is automatically measured to assess anxiety behavior.

### Neuroimaging

All neuroimaging studies were performed using a 7 Tesla/16 cm Bruker Pharmascan or a 7 Tesla 21 cm Bruker Biospec (Karlsure, Germany) MRI/MRS system. Prior to all scans, mice were anesthetized by inhalation of 1-2% isoflurane in a nitrous oxide/oxygen mixture. The animals were subsequently placed into a custom animal holder equipped with a receive only surface coil. Data were acquired using volume transmit and surface coil receive. Respiratory monitoring, gating of the MRI system, and temperature monitoring were done using an SA instruments model 1025 MRI compatible physiological monitoring system (Model 1025, SA Instruments, Stony Brook, NY).

### ^1^H MRS acquisitions

Single voxel localized spectra were acquired in the cerebral cortex (Figure 4A) and cerebellum (Figure 4C) using point resolved spectroscopy (PRESS) with outer volume suppression and high bandwidth pulses to optimize sequence performance. Spectra were acquired with a repetition time of 4 seconds, echo time of 50 ms, 320 averages.

### Spectroscopic analyses

**Figure 10:**
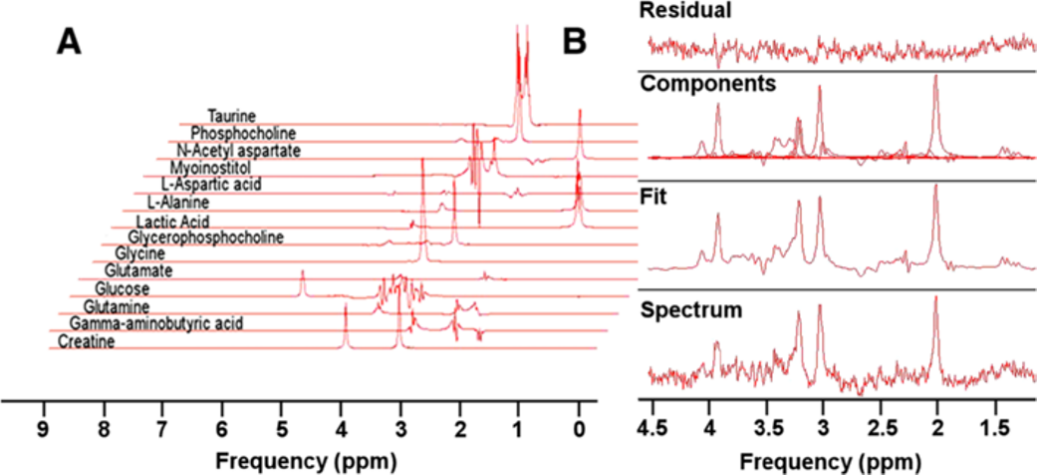
^**1**^ **H MR spectrum and fit. A**: Basis set showing the spectra used to deconvolute the individual components in the in-vivo localized PRESS spectrum. **B**: In vivo spectrum, fit individual components, and the difference between the fit and the original spectrum (residual) from a single acquisition located in the cerebral cortex.

Spectroscopic data were processed by removal of residual water signal using the HLVSD filter. Spectra from ^1^H MRS data sets were curve fit in the time domain using the QUEST algorithm [68, 69] in jMRUI version 4.0 (http://www.mrui.uab.es/mrui), which fits results to a sum of individual metabolite spectra (basis set, Figure 10A) to determine metabolite concentrations (Figure 10B) [22]. Results were expressed as a percentage of the sum of all 14 metabolites (alanine, aspartic acid, creatine, gamma-aminobutyric acid, glutamine, glucose, glutamate, glycine, glycerophosphocholine, lactic acid, myoinostitiol, NAA, phosphocholine and taurine) as a semi-quantitative method for reporting metabolite concentrations in institutional units (I.U.). Glycerophosphocholine and phosphocholine were added and reported as total choline containing compounds.

### DTI

DTI data were obtained using an actively decoupled 72 mm volume coil transmitter and a laboratory built 1.25 × 1.5 cm surface coil receiver. Reference coronal mouse brain images were obtained using 3D FLASH with acquisition parameters of 24 × 24 × 16 mm field-of-view (FOV), a 128 × 128 × 32 matrix, 35° flip angle, 50 ms repetition time (TR), 3 ms echo time (TE), and two averages for a total acquisition time of 7.5 min. DTI data were acquired using single-shot diffusion-weighted spin-echo echo-planar imaging (TE = 43 ms). Respiratory gating was used to prevent motion artifacts. Acquisition was repeated at TR = 10–15 s depending on respiratory rate and one shot per breath. EPI acquisition parameters included: 14 slices, 200 kHz bandwidth, 96 × 96 in-plane acquisition zero-filled to 256 × 256, and a 0.5-mm slice thickness. The diffusion encoding used was a balanced, rotationally invariant and alternating polarity icosahedral scheme (12 directions) as detailed in our previous work [70].

Analyses of the diffusion-weighted data were performed using custom programs written in IDL as previously described [71–73]. Analyses produced maps of the tensor diffusivities (λ_1_, λ_2_, λ_3_), mean diffusivity (*D*_av_) where: *D*_av_ = 1/3*( λ_1_ + λ_2_ + λ_3_) and fractional anisotropy (FA), where: . Transverse (λ_⊥_ = (λ_2_ + λ_3_)/2) and longitudinal (λ_∥_ = λ_1_) components of the diffusion tensor were obtained as previously described [74].

### Histopathology

For immunocytochemical analyses mouse tissues were post-fixed for 24 hours in 4% paraformaldehyde (PFA) and embedded in paraffin. Brain sections were selected from the corresponding regions analyzed by DTI. Five micron paraffin embedded brain sections were stained with mouse monoclonal antibodies to 200 and 68 kDa neurofilaments (NF) (clone 2 F11; 1:200; Dako), synaptophysin (SYN) (clone SY38; 1:1000; EMD Millipore, Temecula, CA), myelin oligodendrocyte glycoprotein [(MOG (D-10): sc-166172; 1:500; Santacruz Biotech, Inc.] and myelin associated glycoprotein [MAG (G-11): sc-166780; 1:500; Santacruz Biotech]. Rabbit polyclonal antibodies were applied to glial fibrillary acidic protein (GFAP, 1:1000; Dako), microtubule-associated protein 2 (MAP2, 1:500; EMD Millipore) and ionized calcium binding adaptor molecule 1 (Iba1; 1:500; Wako chemicals). The signals were captured with secondary anti- mouse and rabbit antibodies conjugated to fluorescent probes Alexa Fluor 488 and Alexa Fluor 594 (1:200; Invitrogen). Nuclei were labeled with 4′, 6-diaminido-2-phenylindole (DAPI). Slides were then cover-slipped with ProLong Gold anti-fade reagent mounting media (Invitrogen, Carlsbad, CA). Images were captured at wavelengths (20 nm intervals between 420 nm to 720 nm) encompassing the emission spectra of the probes using separate fluorescent filter blocks (DAPI, FITC HYQ, Texas Red HYQ; Nikon Instruments, Inc.), with 20× and 40× objectives. The fluorescence emission of each probe and autofluorescence of the tissue samples were analyzed by the multispectral imaging/image analysis using a Nuance EX camera [22] fixed to a Nikon Eclipse E800 using Nuance software (Cambridge Research and Instrumentation, Woburn, MA). Two to four images per region were obtained for each protein from two to four brain sections/mouse. Results were averaged from each individual area for each antigen to represent density and used as a single result per mouse for statistical analysis. The design for statistical analysis consisted of one between subject factor considered as a fixed effect and evaluated with a random effects ANOVA model to account for the anticipated correlated data due to multiple observations from each animal. Differences between infected and uninfected brain staining are illustrated in Figure 4B. The quantitation data were expressed as average ± SEM of signal counts relative fluorescence units/ μm^2^.

### Statistical analyses

Statistical analyses were performed in SAS 9.4 (SAS Institute, Inc, Cary, N.C.). The design consisted of one between factor (infected/control) and one within factor (time, consisting of 5 measurement collected over 4-week intervals). The data were evaluated with a repeated measures ANOVA model that applied an autoregressive covariance structure to account for the correlated nature of the data. Time and treatment were entered as fixed effects and their main effects and interactions were evaluated. Average DTI measures for the infected versus the uninfected control group were compared at each of the five time points (0, 4, 8, 12, 16 weeks). Within each group, the measurements at times 4, 8, 12, and 16 were compared to week 0. To account for multiple comparisons among the group means, adjustments to the p-values and confidence intervals for the differences were computed with simulation techniques, the recommended method for adjustments due to multiplicity for the repeated measures ANOVA model [75]. The means with confidence intervals were then depicted with graphs, which indicate whether a significance difference exists between the two group means at each time point (*) and the difference of the means for week with the pre-infection time for each group (^). Histological results provided with up to eight response variables in each section. The design consists of one between subject factors (HIV infected compared to uninfected controls), treated as a fixed effect. Data existing in a section for the response variables contained from 2 to 4 measurements collected from each subject. The data were evaluated with a random effects ANOVA model to account for the correlation within each mouse. Correlations were performed using Spearman correlations with exact p-values to account for small numbers of samples. All statistical significance tests were two-sided. All statistical analyses were generated with PROC MIXED and PROC FREQ from SAS/STAT. Behavioral data obtained from OFA were analyzed using SAS/STAT and statistical significance was calculated using post-hoc t-test. Graphs were produced with Microsoft Excel software. A *p* less than 0.05 was considered significant.

## Electronic supplementary material

Additional file 1: Figure S1: Identification of HIV-1 infected human cells in spleen and brain. A. Five micron thick paraffin-embedded tissue sections were double florescent stained for human CD14 (macrophages) and HIV-1p24 (viral core protein, top panels) or human CD4 (T lymphocytes) and HIV-1p24 on the bottom panels. Merged pictures show HIV-1 infected (pictured in yellow) human CD14 or CD4 positive cells, original magnification 400**×**. B. Sections of brain tissues were peroxidase-stained for HLA-DR (human immunocytes) in meninges, brain parenchyma and perivascular spaces. HIV-1p24 cells are pictured on corresponding adjacent sections. Top insets show a magnified view of identified cells (see arrows). Top panel shows a HIV-1p24 cell simultaneously stained for human CD163 (macrophages). C. Sections showing perivascular human CD163 positive macrophages. B and C are at original magnification 200× and insets at 1000×. (DOCX 8 MB)

Additional file 2: Figure S2: Complete set of histology results comparing uninfected humanized mice (green, n = 10) to HIV-1 infected controls (red, n = 20) from A: M2 region of the cerebral cortex, B: Whisker barrel region of the cerebral cortex, C: Corpus callosum, D: CA1 region of the hippocampus, E: CA2 region of the hippocampus, F: CA3 region of the hippocampus, G: Dentate gyrus, H: Cerebellum and I: Brainstem. *Significant differences (p < 0.05). (DOCX 728 KB)

Additional file 3: Figure S3: Cerebral Cortex Metabolite Levels (Means ± SEM) expressed as a percentage of total signal contribution from ^1^H MRS scans of (red) HIV-1 infected humanized mice (n = 7) and (black) uninfected humanized mice (n = 7) over time. Time zero in infected mice is preinfection with subsequent spectra acquired every four weeks up to 16 weeks in both infected and uninfected mice. *p < 0.05 control vs infected mice, ^p < 0.05 vs time zero in control mice, (red “^” symbol) p < 0.05 vs preinfection in infected mice. (DOCX 459 KB)

Additional file 4: Figure S4: Cerebellum Metabolite Levels (Means ± SEM) expressed as a percentage of total signal contribution from ^1^H MRS scans of (red) HIV-1 infected humanized mice (n = 7) and (black) uninfected humanized mice (n = 7) over time. Time zero, in infected mice is preinfection with subsequent spectra acquired every four weeks up to 16 weeks in both infected and uninfected mice. *p < 0.05 control vs infected mice, ^p < 0.05 vs time zero in control mice, (red “^” symbol) p < 0.05 vs preinfection in infected mice. (DOCX 457 KB)

Additional file 5: Figure S5: Comparison of DTI metrics (mean ± SEM) in CA1, CA2, CA3, and Dentate Gyrus (from left to right) as shown in Figure 7 in uninfected (black, n = 7) and HIV-1 infected (red, n = 8) humanized mice. Shown are (from top to bottom) Mean diffusivity (D_av_), Fractional anisotropy (FA), transverse component of diffusivity ((λ_t_), and longitudinal component of diffusivity ((λ_l_). (red “*” symbol) p < 0.05 control vs infected mice, ^t^p < 0.05 vs time zero in control mice, (red “^” symbol) p < 0.05 vs preinfection in infected mice. (DOCX 612 KB)

Additional file 6: Figure S6: Comparison of DTI metrics (mean ± SEM) in frontal cortex, middle cerebral cortex, M2 region of cerebral cortex, and whisker barrels (from left to right) as shown in Figure 7 in uninfected (black, n = 7) and HIV-1 infected (red, n = 8) humanized mice. Shown are (from top to bottom) Mean diffusivity (D_av_), Fractional anisotropy (FA), transverse component of diffusivity (λ_t_), and longitudinal component of diffusivity (λ_l_). (red “*” symbol) p < 0.05 control vs infected mice, ^t^p < 0.05 vs time zero in control mice, (red “t” symbol) p < 0.05 vs preinfection in infected mice. (DOCX 685 KB)

Additional file 7: Figure S7: Comparison of DTI metrics over time (mean ± SEM) from left to right in the Genu of the Corpus Callosum (CC) and the Splenium of the CC, as shown in Figure 7. Results are shown from uninfected (black, n = 7) and HIV-1 infected (red, n = 8) humanized mice. Shown are (from top to bottom) Mean diffusivity (D_av_), Fractional anisotropy (FA), transverse component of diffusivity (λ_t_), and longitudinal component of diffusivity ((λ_l_). (red “*” symbol) p < 0.05 control vs infected mice, ^t^p < 0.05 vs time zero in control mice, (red “t” symbol) p < 0.05 vs preinfection in infected mice. (DOCX 347 KB)

## Abbreviations

ART: Antiretroviral therapy
CNS: Central nervous system
cART: Combination antiretroviral therapy
DTI: Diffusion tensor magnetic resonance imaging
FACS: Fluorescence-activated cell sorting
FA: Fractional anisotropy
GABA: Gamma-amino butyric acid
GFAP: Glial fibrillary acidic protein
HAND: HIV-associated neurocognitive disorders
Iba-1: Ionized calcium binding adaptor molecule 1
λ_l_: Longitudinal Diffusivity
M2c: M2 region of the cerebral cortex
D_av_: Mean diffusivity
MAP2: Microtubule-associated protein 2
MAG: Myelin associated glycoprotein
MOG: Myelin oligodendrocyte glycoprotein
NAA: N-acetyl aspartate
NF: Neurofilament protein
NSG: NOD/*scid*-IL-2Rg_c_^*null*^
OFA: Open field activity
^1^H MRS: Proton magnetic resonance spectroscopy
SYN: Synaptophysin
TCID_50_: Tissue culture infective dose 50
λ_t_: Transverse diffusivity
VL: Viral load.
