# Supplementary material for: Associations between brain microstructures, metabolites, and cognitive deficits during chronic HIV-1 infection of humanized mice
Source: Mol Neurodegener. 2014 Dec 18;9:58. doi: 10.1186/1750-1326-9-58 (PMC4297430; doi:10.1186/1750-1326-9-58)
Supplement: Supplementary file 1 — Additional file 1: Figure S1: Identification of HIV-1 infected human cells in spleen and brain. A. Five micron thick paraffin-embedded tissue sections were double florescent stained for human CD14 (macrophages) and HIV-1p24 (viral core protein, top panels) or human CD4 (T lymphocytes) and HIV-1p24 on the bottom panels. Merged pictures show HIV-1 infected (pictured in yellow) human CD14 or CD4 positive cells, original magnification 400×. B. Sections of brain tissues were peroxidase-stained for HLA-DR (human immunocytes) in meninges, brain parenchyma and perivascular spaces. HIV-1p24 cells are pictured on corresponding adjacent sections. Top insets show a magnified view of identified cells (see arrows). Top panel shows a HIV-1p24 cell simultaneously stained for human CD163 (macrophages). C. Sections showing perivascular human CD163 positive macrophages. B and C are at original magnification 200× and insets at 1000×. (DOCX 8 MB) [file 13024_2014_569_MOESM1_ESM.docx]

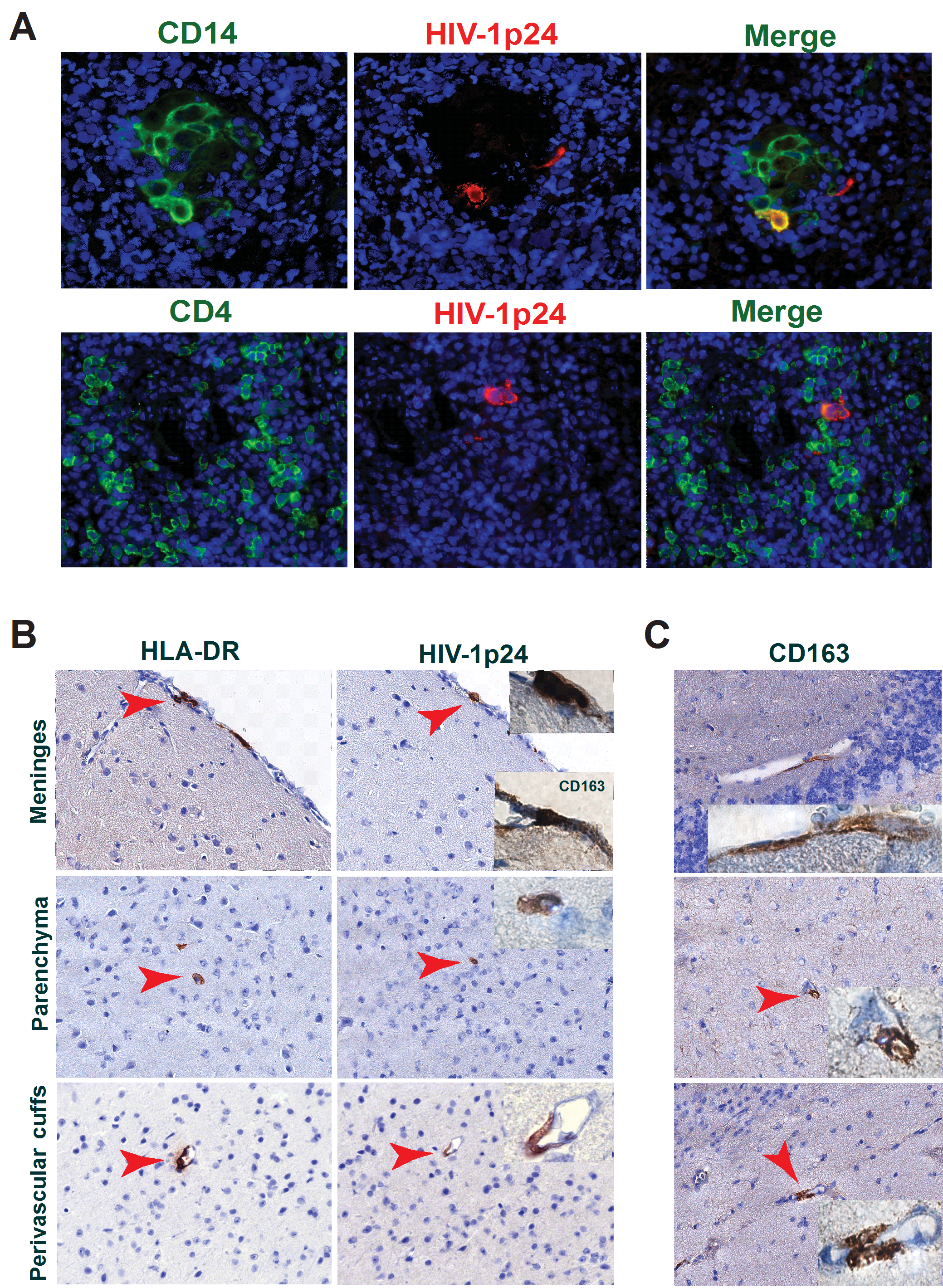


Supplemental Figure 1: Identification of HIV-1 infected human cells in spleen and brain. A. Five micron thick paraffin-embedded tissue sections were double florescent stained for human CD14 (macrophages) and HIV-1p24 (viral core protein, top panels) or human CD4 (T lymphocytes) and HIV-1p24 on the bottom panels. Merged pictures show HIV-1 infected (pictured in yellow) human CD14 or CD4 positive cells, original magnification 400x. B. Sections of brain tissues were peroxidase-stained for HLA-DR (human immunocytes) in meninges, brain parenchyma and perivascular spaces. HIV-1p24 cells are pictured on corresponding adjacent sections. Top insets show a magnified view of identified cells (see arrows). Top panel shows a HIV-1p24 cell simultaneously stained for human CD163 (macrophages). C. Sections showing perivascular human CD163 positive macrophages. B and C are at original magnification 200x and insets at 1000x.
