# Supplementary material for: Associations between brain microstructures, metabolites, and cognitive deficits during chronic HIV-1 infection of humanized mice
Source: Mol Neurodegener. 2014 Dec 18;9:58. doi: 10.1186/1750-1326-9-58 (PMC4297430; doi:10.1186/1750-1326-9-58)
Supplement: Supplementary file 2 — Additional file 2: Figure S2: Complete set of histology results comparing uninfected humanized mice (green, n = 10) to HIV-1 infected controls (red, n = 20) from A: M2 region of the cerebral cortex, B: Whisker barrel region of the cerebral cortex, C: Corpus callosum, D: CA1 region of the hippocampus, E: CA2 region of the hippocampus, F: CA3 region of the hippocampus, G: Dentate gyrus, H: Cerebellum and I: Brainstem. *Significant differences (p < 0.05). (DOCX 728 KB) [file 13024_2014_569_MOESM2_ESM.docx]

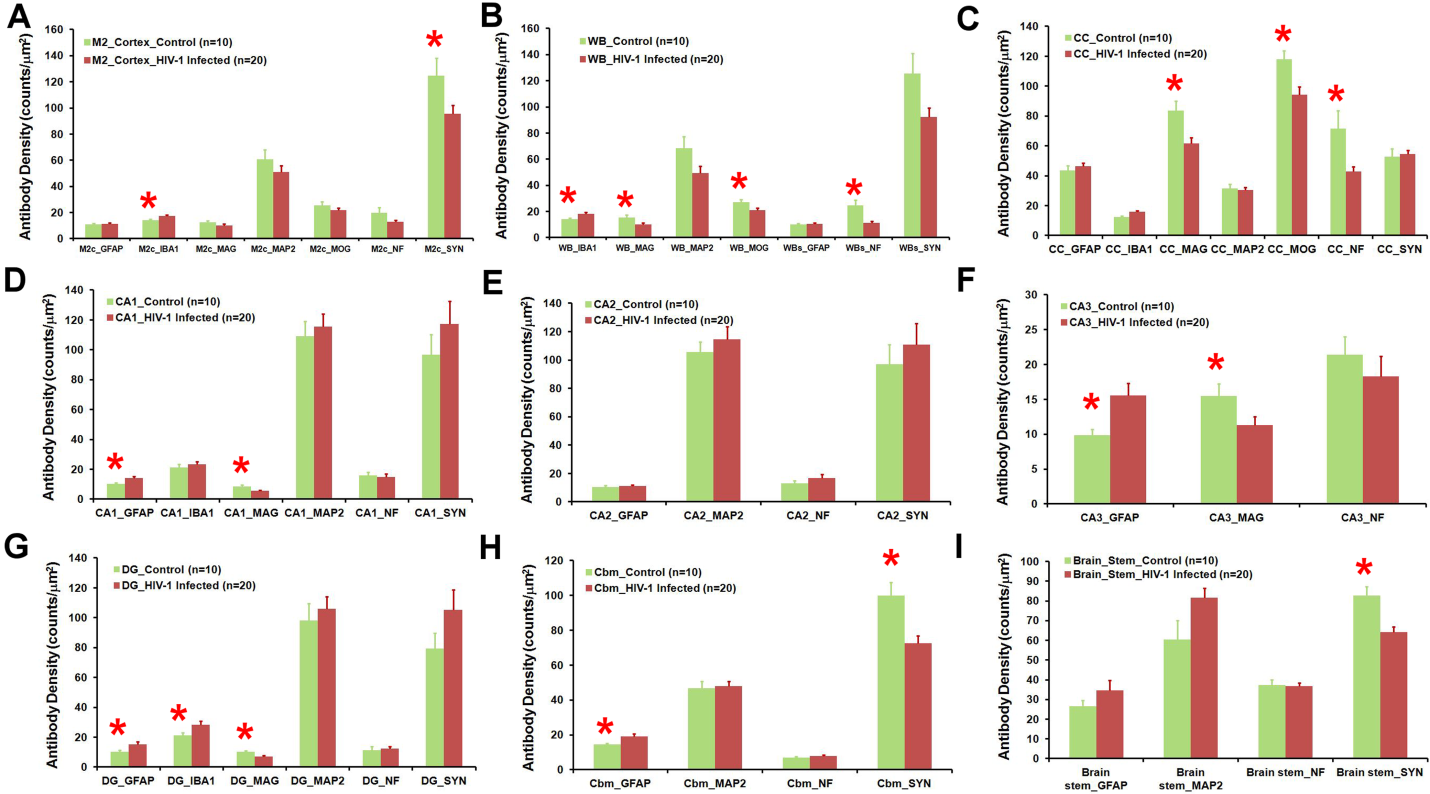


Supplementary Figure 2. Complete set of histology results comparing uninfected humanized mice (green, n=10) to HIV-1 infected controls (red, n=20) from A: M2 region of the cerebral cortex, B: Whisker barrel region of the cerebral cortex, C: Corpus callosum, D: CA1 region of the hippocampus, E: CA2 region of the hippocampus, F: CA3 region of the hippocampus, G: Dentate gyrus, H: Cerebellum and I: Brainstem. *Significant differences (p<0.05).
