# Supplementary material for: Associations between brain microstructures, metabolites, and cognitive deficits during chronic HIV-1 infection of humanized mice
Source: Mol Neurodegener. 2014 Dec 18;9:58. doi: 10.1186/1750-1326-9-58 (PMC4297430; doi:10.1186/1750-1326-9-58)
Supplement: Supplementary file 4 — Additional file 4: Figure S4: Cerebellum Metabolite Levels (Means ± SEM) expressed as a percentage of total signal contribution from 1H MRS scans of (red) HIV-1 infected humanized mice (n = 7) and (black) uninfected humanized mice (n = 7) over time. Time zero, in infected mice is preinfection with subsequent spectra acquired every four weeks up to 16 weeks in both infected and uninfected mice. *p < 0.05 control vs infected mice, ^p < 0.05 vs time zero in control mice, (red “^” symbol) p < 0.05 vs preinfection in infected mice. (DOCX 457 KB) [file 13024_2014_569_MOESM4_ESM.docx]

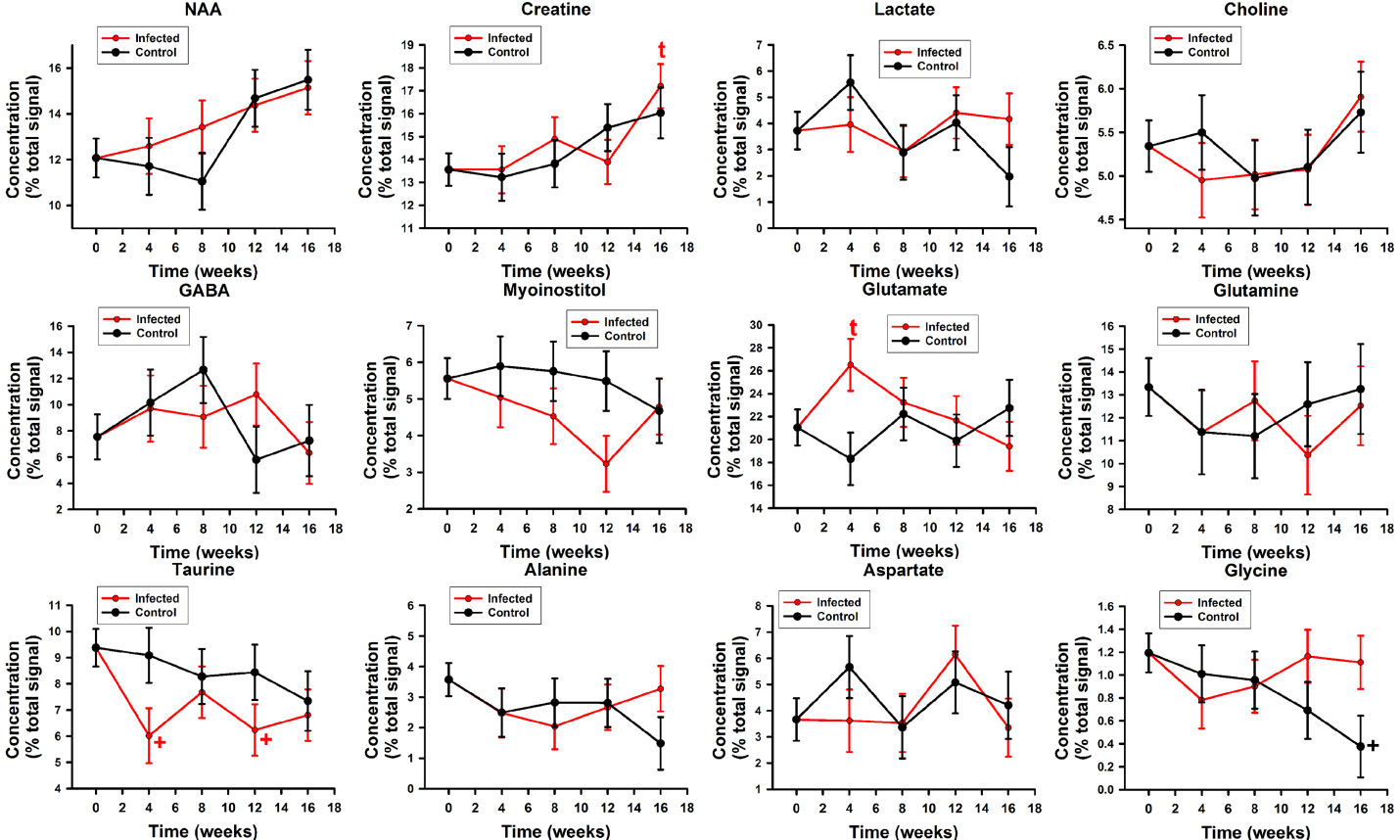


Supplementary Figure 4. Cerebellum Metabolite Levels (Means ± SEM) expressed as a percentage of total signal contribution from ^1^H MRS scans of (red) HIV-1 infected humanized mice (n=7) and (black) uninfected humanized mice (n=7) over time. Time zero, in infected mice is preinfection with subsequent spectra acquired every four weeks up to 16 weeks in both infected and uninfected mice. *p<0.05 control vs infected mice, ^p<0.05 vs time zero in control mice, ^p<0.05 vs preinfection in infected mice.
