# Supplementary material for: Associations between brain microstructures, metabolites, and cognitive deficits during chronic HIV-1 infection of humanized mice
Source: Mol Neurodegener. 2014 Dec 18;9:58. doi: 10.1186/1750-1326-9-58 (PMC4297430; doi:10.1186/1750-1326-9-58)
Supplement: Supplementary file 5 — Additional file 5: Figure S5: Comparison of DTI metrics (mean ± SEM) in CA1, CA2, CA3, and Dentate Gyrus (from left to right) as shown in Figure 7 in uninfected (black, n = 7) and HIV-1 infected (red, n = 8) humanized mice. Shown are (from top to bottom) Mean diffusivity (Dav), Fractional anisotropy (FA), transverse component of diffusivity ((λt), and longitudinal component of diffusivity ((λl). (red “*” symbol) p < 0.05 control vs infected mice, tp < 0.05 vs time zero in control mice, (red “^” symbol) p < 0.05 vs preinfection in infected mice. (DOCX 612 KB) [file 13024_2014_569_MOESM5_ESM.docx]

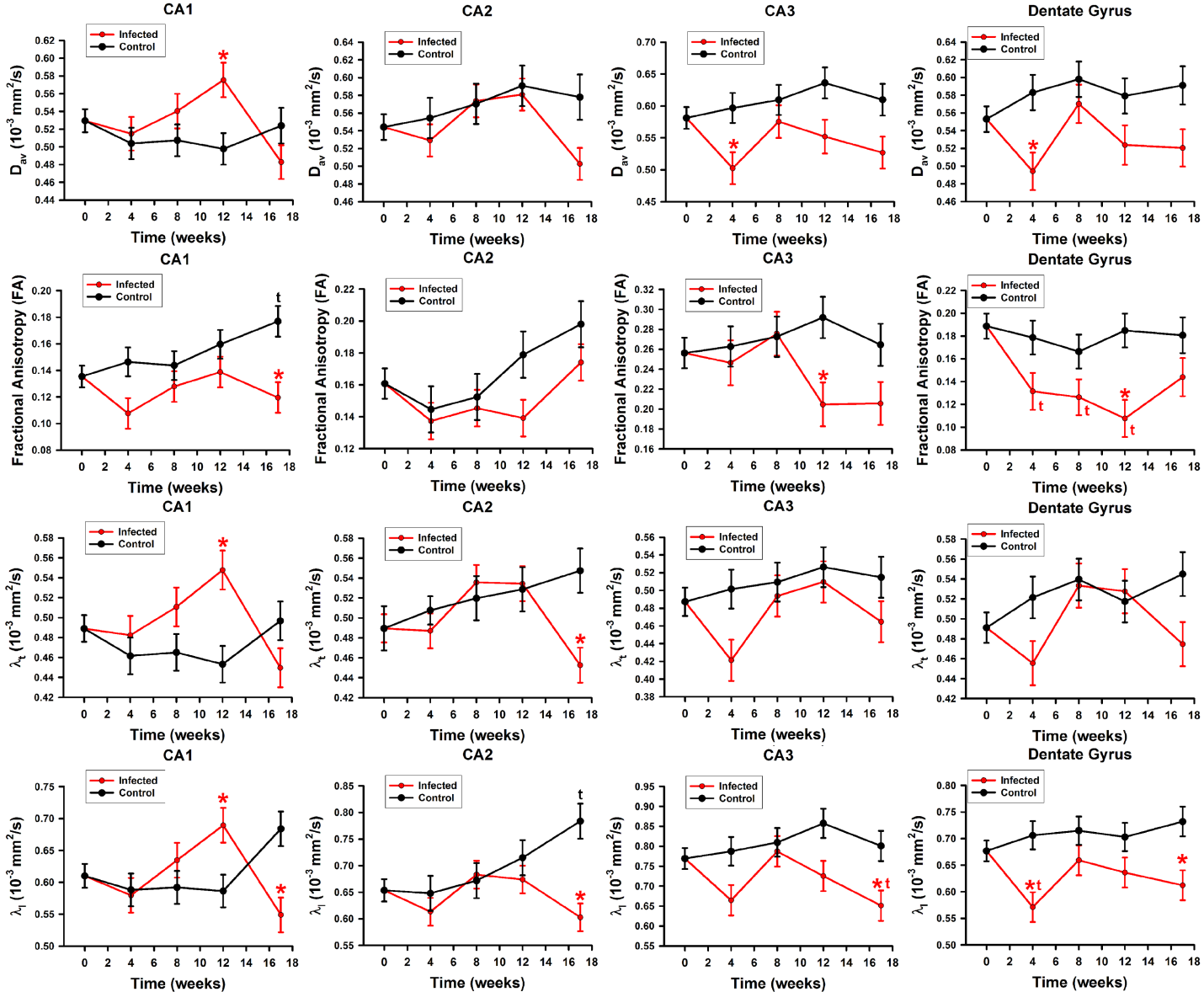


Supplementary Figure 5. Comparison of DTI metrics (mean ± SEM) in CA1, CA2, CA3, and Dentate Gyrus (from left to right) as shown in Figure 7 in uninfected (black, n=7) and HIV-1 infected (red, n=8) humanized mice. Shown are (from top to bottom) Mean diffusivity (D_av_), Fractional anisotropy (FA), transverse component of diffusivity (λ_t_), and longitudinal component of diffusivity (λ_l_). *p<0.05 control vs infected mice, ^t^p<0.05 vs time zero in control mice, ^t^p<0.05 vs preinfection in infected mice.
